# Mineral-doped quantum fertilizers: boron-doped carbon dots promote sustainable agriculture and bacterial disease management

**DOI:** 10.1039/d6ra00122j

**Published:** 2026-06-04

**Authors:** Sourav Chakraborty, Suresh K. Mondal, Piyush Baindara, Tarun K. Barik, Souri Roy, Debashis Panda, Gourisankar Ghosh, Santi M. Mandal

**Affiliations:** a Department of Bioscience and Biotechnology, Indian Institute of Technology Kharagpur Kharagpur 721302 WB India smmandal@iitkgp.ac.in smmandal@ucsd.edu; b Animal Science Research Center, Division of Animal Sciences, University of Missouri Columbia MO 65211 USA; c Department of Physics, Achhruram Memorial College, Jhalda Purulia 723202 WB India; d Department of Biotechnology, Sister Nivedita University Kolkata 700156 India; e Faculty of Engineering and Technology, Sri Sri University Cuttack Odisha 754006 India; f Department of Biochemistry and Molecular Biophysics, University of California 9500 Gilman Dr, La Jolla 92093 San Diego CA USA

## Abstract

Mineral (micro- and macronutrient)-doped quantum fertilizers developed *via* green synthesis represent a novel and transformative paradigm in sustainable agriculture, offering an ecofriendly strategy to maximize nutrient use efficiency, stimulate plant growth, and significantly reduce dependence on conventional chemical fertilizers. Here, we report the synthesis of Mg-, Zn-, Fe- and B-doped carbon quantum dots *via* a microwave-assisted method using oxalic acid as a molecular precursor. Notably, boron-doped carbon quantum dots (B-QDs) exhibit dual roles of plant growth promotion and significant antibacterial activity, as comprehensively characterized and functionally validated. Structural analyses (UV-vis spectroscopy, TEM, XPS, and fluorescence spectroscopy) confirmed the formation of quantum dots with an average diameter of 8–12 nm. B-QD treatments significantly enhanced seed germination, root elongation, and root number compared with the control (without mineral), boron alone, and Mg-doped and Fe-doped carbon quantum dots (Mg-QDs and Fe-QDs, respectively). However, Zn-doped carbon quantum dots (Zn-QDs) showed better seed germination and root elongation ability, but no antibacterial activity was observed. Fluorescence imaging further demonstrated efficient internalization and distribution of B-QDs in root tissues, while binding studies revealed strong interactions with pectic acid, a major polysaccharide in the cell walls suggesting a role in modulating cell wall dynamics. Antibacterial assays confirmed selective inhibition of phytopathogens such as *Pseudomonas syringae* and *Ralstonia solanacearum* without adversely affecting beneficial soil microbes. Furthermore, B-QDs displayed potent antibiofilm activity, validating their dual function as both plant growth promoters and eco-friendly antimicrobial agents. These seminal findings catalyze a new scientific quest to harness the power of “quantum fertilizers” toward advanced sustainable agriculture and next-generation plant disease management.

## Abstract 2

Boron-doped carbon quantum dots exhibit dual functionality by promoting plant growth and providing potent antimicrobial activity against phytopathogens.

## Graphical abstract

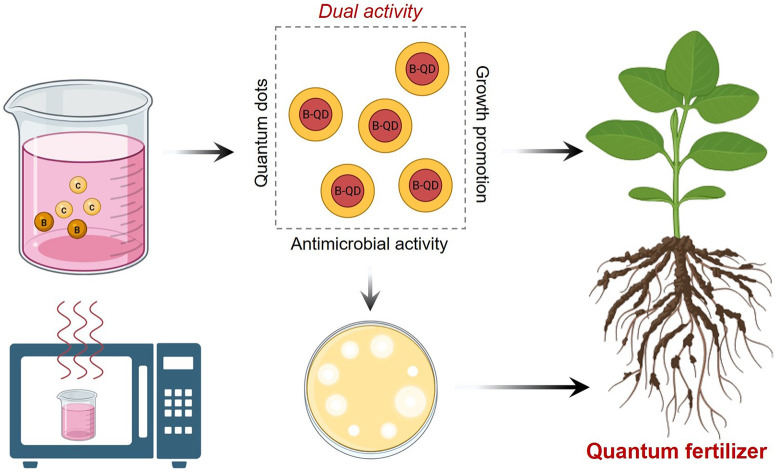

## Introduction

Essential elements serve critical roles in plant nutrition by participating in metabolic, structural, and physiological processes. Macronutrients like nitrogen, phosphorus, and potassium are vital for protein synthesis, energy transfer, and osmoregulation,^1^ while micronutrients such as boron, iron, and zinc function as cofactors in enzymatic reactions and are crucial for hormonal regulation, photosynthesis, and reproductive development.^2^ Among essential micronutrients, boron plays a pivotal role in plant growth and development, being indispensable for cell wall integrity, membrane function, reproductive processes, and root elongation.^3^

In general, plants absorb ionic and molecular forms of elements that are water-soluble and can be transported through roots and plant tissues. The availability of these forms in the rhizosphere greatly influences plant growth, development, and stress tolerance.^4^ Plants can absorb elements in their nanoform, including nanoparticles (NPs) and nanostructured formulations,^5^ but the mechanism, efficiency, and impact depend on several factors like particle size, charge, solubility, surface chemistry, and plant species. Nanofertilizers enhance nutrient use efficiency by enabling controlled release, targeted delivery, and reduced nutrient losses, leading to improved plant growth and reduced environmental pollution.^6,7^ Their nanoscale size allows better root penetration and uptake, minimizing the overuse of conventional fertilizers. In 2019, we summarized the impact of nanofertilizers in sustainable agriculture.^8^ However, poorly controlled particle size in nanoparticles in agriculture often exhibit reduced uptake efficiency and limited mobility in plant tissues due to size-related exclusion and agglomeration.^6^ They may also cause phytotoxic effects and disrupt the soil's microbial balance when applied in excess.^9^

To overcome the limitations of nanofertilizers, quantum fertilizers, prepared by the use of quantum particles of the same elements, have been tested. Quantum dots are a specialized subset of nanoparticles distinguished by their quantum behavior.^10^ Nanoparticles are particles with sizes between 1 and 100 nm that may or may not exhibit quantum effects, while quantum particles (quantum dots) are smaller (<10 nm) semiconductor nanoparticles that display size-dependent quantum confinement and unique optical properties.^11^ In contrast, quantum dots are typically semiconductor nanocrystals with a neutral overall charge but can exhibit surface ionic groups or ligands that influence their solubility and stability.^10,11^

In this study, we report the synthesis and characterization of boron-doped quantum dots (B-QDs) and evaluate their role in promoting plant growth. The unique physicochemical properties of B-QDs not only enable efficient boron delivery to plants but also allow potential tracking of nutrient uptake due to their intrinsic optical behaviour. We investigate the effects of B-QDs on seed germination, root and shoot development, antibacterial properties against soil pathogens, and antioxidant response in *Vigna mungo* crop systems. Our findings present a novel approach to leveraging the boron element in the form of quantum dots, with implications for sustainable farming and disease management.

## Results and discussion

### Antimicrobial and plant growth-promoting activities of boron-doped carbon quantum dots (B-QDs)

**Fig. 1:**
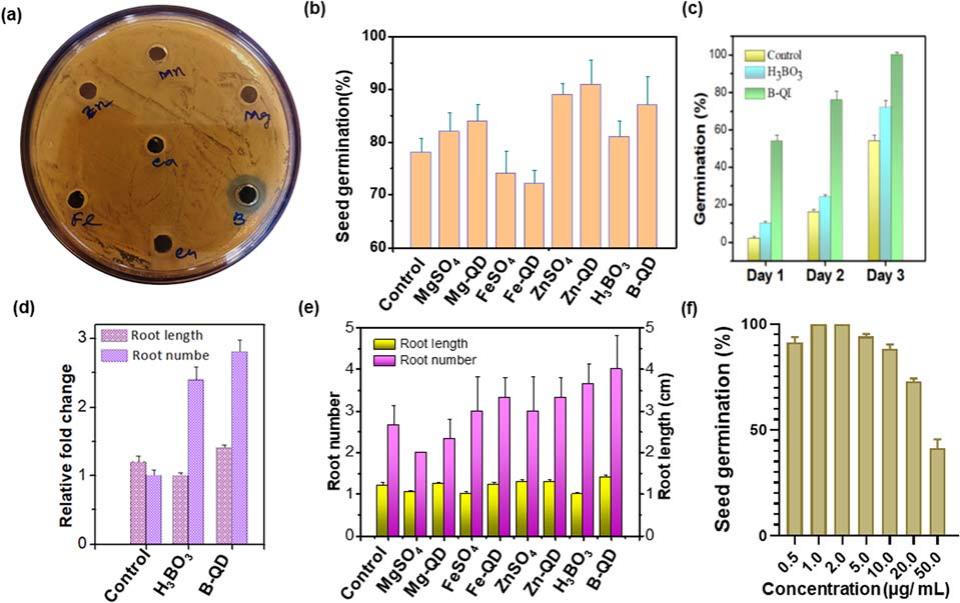
Antimicrobial and plant growth-promoting activities of boron-doped carbon quantum dots and mineral-doped QDs. (a) Agar well diffusion assay showing the antimicrobial activity of different elements (Zn, Mn, Mg, Fe, Ca, B, and Cu), with B-QDs producing a distinct inhibition zone. (b) Bar graph showing the comparative seed germination percentage of different treatments of micronutrients and their corresponding particles against *Vigna mungo*. (c) Seed germination assay of *V. mungo* under control, boric acid (H_3_BO_3_), and B-QD treatments, showing significantly higher germination with B-QDs across 3 days. (d) Relative fold change in the root length and root number of seedlings treated with B-QDs compared to the controls, indicating enhanced root development. (e) Comparative analysis of the shoot and root biomass (fresh and dry weight) under different treatments, where the B-QD treatment shows the highest growth promotion. (f) Effect of different concentrations of B-QDs on seed germination.

The antibacterial activity of different mineral-doped carbon quantum dots (QDs) was assessed against *Pseudomonas syringae* using the agar well diffusion method (Fig. 1a). Distinct zones of inhibition were observed around the wells treated with boron (B)-doped carbon QDs. In contrast, QDs derived from Zn, Mn, Mg, Ca, Fe, and Cu exhibited little to no inhibition against *P. syringae* under the tested conditions. The control well showed no inhibitory effect, confirming that the observed activity was due to the specificity of B-QDs. This is evidence of boron's special antimicrobial property, originating from its role in impairing the integrity of bacterial cell walls and metabolic processes.^19^

To check whether the seed germination performance was enhanced by the application of mineral-doped carbon QDs, studies were performed, and the treatments with ZnSO_4_ and Zn-QDs displayed the highest values, which were noticeably higher than those of the untreated control. Treatments with FeSO_4_ and Fe-QDs, by contrast, had the lowest responses, suggesting that the dose or exposure level can vary (Fig. 1b). These results are in line with previous studies on nanoparticle-mediated growth promotion and imply that B-QDs can function as effective plant growth stimulants, increasing physiological activity and biomass accumulation.^20^ Throughout the three days, the germination rate of seeds treated with B-QDs was higher than that of the control and H_3_BO_3_ treatments (Fig. 1c). Germination in the B-QD group was nearly 100% by day 3, which was a substantial improvement compared to H_3_BO_3_ (∼70%) and control (∼55%) treatments. According to these findings, B-QDs have a better stimulatory effect on early seed germination, most likely because of their increased bioavailability and effective engagement with cellular metabolism.^21^

In the study measuring root number and length, compared with the control, there was a noticeable increase in both the root length and number in the B-QD treatment. The greatest response was seen in the number of roots, which increased by almost three times, and in the length of the roots, which increased by more than two times (Fig. 1d).

Compared to the untreated control and several QDs, the root characteristics gradually increased for the treatments with B-QDs (Fig. 1e). These enhancements might result from the boron-containing nanodots' capacity to interact with cell walls' pectic polysaccharides, which promotes root meristem cell division and elongation. Despite their beneficial role at low doses, at higher concentrations, B-QDs induced significant phytotoxicity by inhibiting seed germination and ultimately impairing their growth (Fig. 1f). The importance of the designed nanostructures in enhancing nutrient acquisition and stress tolerance is supported by the similar growth-promoting effects of nanomaterials on the plant root architecture.

### Optical and structural characterization of B-QDs

**Fig. 2:**
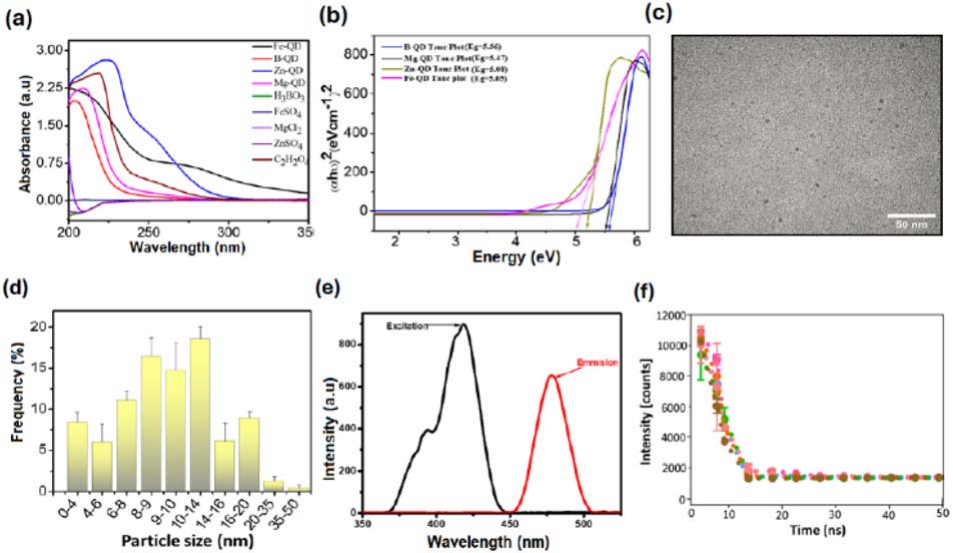
Optical and structural characterization of boron-doped carbon quantum dots (B-QDs). (a) UV-visible absorption spectra of micronutrients and their corresponding particle formation. (b) Tauc plots of different quantum dots: Mg-QDs, Fe-QDs, B-QDs and Zn-QDs. (c) Transmission electron microscopy (TEM) image showing well-dispersed, spherical B-QDs with a uniform distribution. (d) Histogram of the particle size distribution of B-QDs, indicating that abundant species are 8–12 nm in size. (e) Photoluminescence excitation and emission spectra of B-QDs, confirming strong fluorescence properties. (f) Time-resolved fluorescence decay profiles of B-QDs under different excitation wavelengths, indicating multiexponential decay and a stable fluorescence lifetime.

The UV-vis absorption spectra of the synthesized mineral-doped carbon quantum dots (Fe-QDs, B-QDs, Zn-QDs, and Mg-QDs) and their corresponding precursor salts (H_3_BO_3_, FeSO_4_, MgCl_2_, ZnSO_4_, and C_2_H_2_O_4_) exhibited distinct features in the UV region (∼250–350 nm) with characteristic absorption tails extending into the visible region, as shown in Fig. 2a. Compared to their respective controls, all QDs showed stronger and broader absorption profiles, confirming the successful formation of nanoscale structures with enhanced light–matter interactions. Among them, B-QDs displayed the most intense absorption peak, indicating efficient photon harvesting and pronounced electronic transitions. Zn-QDs and Mg-QDs also exhibited well-defined absorption, consistent with moderate confinement effects, while Fe-QDs showed relatively lower absorbance, likely due to reduced oscillator strength or a larger effective particle size, which may produce several vibrational and rotational states of a molecule, which lead to separate energy band gaps of slightly different energies. These results highlight the significant role of the precursor chemistry and composition in modulating the optical behaviour of the QDs, consistent with earlier reports on the size- and composition-dependent tunability of semiconductor nanostructures.^22,23^ Furthermore, the optical direct band gaps (*E*_g_) of the biodegradable mineral-doped carbon quantum dots, such as Mg-QDs, Fe-QDs, B-QDs, and Zn-QDs, were determined using Tauc plots derived from the UV-vis absorption spectra by linear extrapolation of the absorption edges using the formula:(*αhν*)^*n*^ = *A*(*hν* − *E*_g_)where *α* is the absorption coefficient, *h* is Planck's constant, *ν* is the incident photon's frequency, *n* is the nature of transmission, and *A* is the energy-independent constant. As shown in Fig. 2b, the calculated band gap energies of the quantum dots were found to be approximately 5.56 eV for B-QDs, 5.47 eV for Mg-QDs, 5.08 eV for Zn-QDs, and 5.05 eV for Fe-QDs. Such high bandgaps indicate the transparent nature of the quantum structures. Among them, B-QDs exhibited the highest band gap, reflecting the strongest quantum confinement and the smallest effective particle size. In contrast, Fe-QDs showed the lowest band gap, suggesting a relatively larger quantum dot size and weaker confinement effects. These variations confirm that the optical properties of the synthesized QDs are highly size- and composition-dependent, demonstrating tunable band structures that are characteristic of quantum-confined nanomaterials.

The TEM micrograph of B-QDs (Fig. 2c) revealed uniformly distributed, nearly spherical nanoparticles without noticeable aggregation, confirming the successful synthesis of well-dispersed nanodots. The high degree of monodispersity and absence of clustering indicated good colloidal stability and controlled nucleation during synthesis. The bulk of the B-QDs were below 10 nm, with the majority falling between 8 and 12 nm, according to the particle size distribution study (Fig. 2d). The strong optical responses shown in UV-vis and Tauc studies are consistent with the creation of ultrasmall nanodots within the quantum-confined domain, which is confirmed by this limited size distribution. B-QDs are interesting candidates for plant growth-promoting nanomaterials because of their homogeneous size, which improves surface reactivity and guarantees dependable bio-interactions.^24^

When stimulated at 360 nm, B-QDs showed a pronounced emission peak at 440 nm, demonstrating robust quantum confinement and effective radiative recombination. The fluorescence spectra (Fig. 2e) of B-QDs showed a sharp emission peak centred around the visible area. The homogeneous particle size distribution suggested by the narrow emission band is consistent with the TEM examination. Fig. 2f shows the time-resolved fluorescence decay spectra of B-QDs obtained at several excitation wavelengths between 300 and 370 nm. The fluorescence lifetime components exhibited a constant decline of about 8 ns, regardless of the excitation fluctuation from 300 to 340 nm.

### XPS analysis of boron-doped carbon quantum dots

**Fig. 3:**
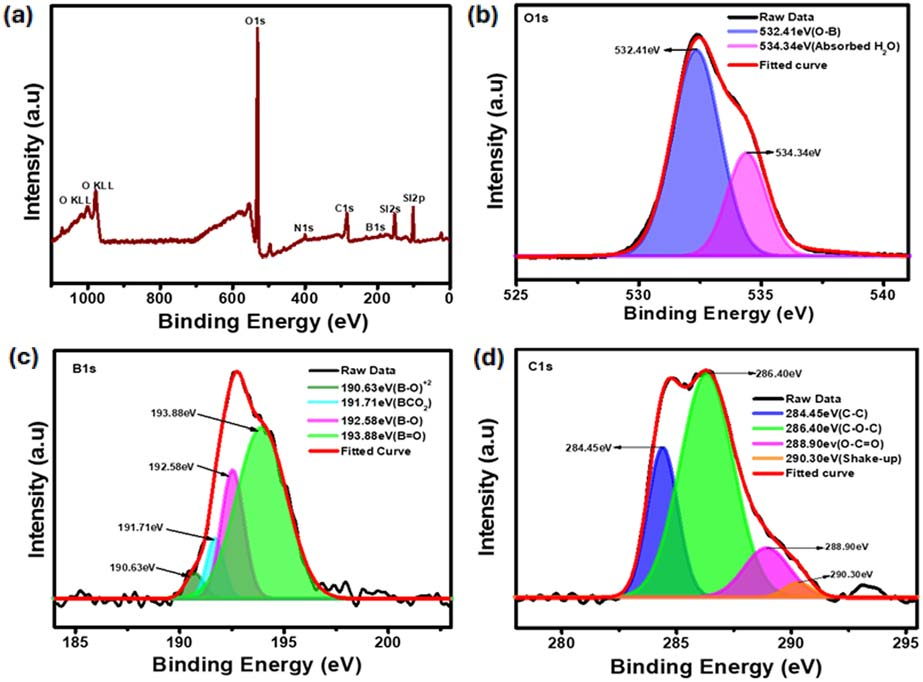
X-ray photoelectron spectroscopy (XPS) analysis of boron-doped carbon quantum dots (B-QDs). (a) Survey XPS spectrum confirming the presence of boron (B), carbon (C), and oxygen (O) elements in B-QDs. (b) High-resolution O 1s spectrum showing characteristic peaks corresponding to C–O, B–O, and surface hydroxyl groups. (d) High-resolution C 1s spectrum deconvoluted into peaks assigned to C–C/C=C, C–O, and O–C=O bonds, indicating surface functionalization. (c) High-resolution B 1s spectrum revealing distinct peaks corresponding to B–O and B–C bonding states, confirming successful boron incorporation into the quantum dot structure.

The XRD spectrum (Fig. 3a) shows a broad peak around 24°, corresponding to the (002) plane of graphitic carbon, confirming the amorphous and turbostratic nature of the B-QDs. The absence of sharp crystalline peaks indicates a small particle size and high disorder, typical of quantum dot structures. The high-resolution C 1s spectrum (Fig. 3d) shows four unique chemical environments. The peak at 284.45 eV indicates graphitic sp^2^-hybridized carbon (C–C/C=C), whereas the peaks at 286.40 eV and 288.90 eV indicate C–O–C and carboxyl functionalities, respectively.^25^ The satellite feature at 290.30 eV indicates the presence of π–π* shake-up transitions, which are common in sp^2^-conjugated carbon systems.^26^ The O 1s spectrum (Fig. 3b) exhibits a primary peak at 532.41 eV, which is indicative of oxygen coordinated to boron (B–O),^27^ as well as a secondary peak at 534.34 eV, which is attributable to physisorbed water. This suggests partial surface hydration or air exposure, which is common in porous or large-surface-area boron-based systems.^28^ The B 1s core-level spectrum (Fig. 3c) is particularly complicated, with four peaks. The signal at 192.58 eV indicates the presence of tricoordinate B–O units,^29^ whereas the peak at 193.88 eV is due to oxidised B=O groups.^30^ The binding energy peak at 191.71 eV indicates the production of boron–carbon–oxygen complexes (*e.g.*, B–CO_2_),^31^ whereas the lowest-energy feature at 190.63 eV could be from (B–O)^2+^ species or partly ionic B–O bonds in defect-rich environments.^32^

### Antibacterial and biofilm eradication efficacy of B-QDs

**Fig. 4:**
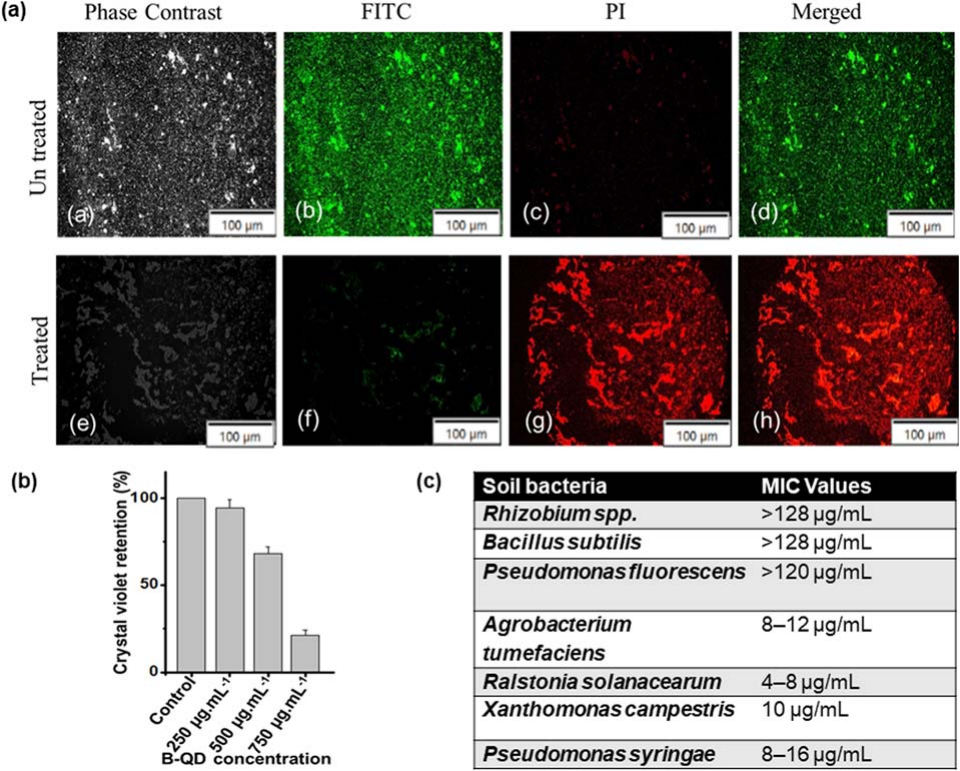
Antibiofilm activity of boron-doped carbon quantum dots (B-QDs). (a) Representative microscopic images showing biofilm formation in the control group, which showed dense and compact bacterial aggregation, whereas B-QD-treated samples exhibited a marked reduction in biofilm thickness, surface coverage, and cell density, indicating strong antibiofilm efficacy. (b) Bar diagram showing the retention of the crystal violet dye as a measure of biofilm biomass. Biofilm formation decreased significantly with increasing concentrations of B-QDs, indicating a dose-dependent inhibition of biofilm growth. (c) Table presenting the minimum inhibitory concentration (MIC) values of B-QDs against different bacterial pathogens.

Antibacterial efficacy against *P. syringae* was verified using B-QDs. Biofilm eradication assays were performed with and without B-QDs. After treatment, cells were stained with LIVE/DEAD™ BacLight™ (Fig. 4a), and the cells treated with B-QDs showed both red and green fluorescence, indicating impaired cell integrity and significant membrane damage compared to the without-B-QD treatment, which displayed only green fluorescence. These findings validate the potent antibacterial potential of B-QDs. After 48 hours of matured biofilm formation, B-QDs demonstrated antibiofilm action against *P. syringae*, and biofilm inhibition was quantified to be 94.32%, 68.21%, and 20.94% at dosages of 128 µg mL^−1^, 64 µg mL^−1^, and 32 µg mL^−1^, respectively (Fig. 4b). The results demonstrated that B-QDs effectively eliminated mature biofilms and had an inhibitory effect on further biofilm formation. A few soil pathogenic bacteria were tested to check the efficacy of B-QDs, and the MIC values were determined against the strains *Ralstonia solanacearum*, *Agrobacterium tumefaciens*, *Xanthomonas campestris*, *Pseudomonas syringae* and *Rhizobium* sp. (Fig. 4c). By contrast, beneficial soil bacteria like *Pseudomonas fluorescens* and *Rhizobium* spp. displayed extremely high MIC values (>120 µg mL^−1^). These strains also showed elevated tolerance to stress conditions, including antimicrobial agents and desiccation. The correlation between high extracellular polysaccharide levels and enhanced resistance suggests that EPS plays a protective role for these bacteria.^33^ The promise of B-QDs as environmentally friendly antimicrobial agents that can target phytopathogens while preserving beneficial soil microorganisms is highlighted by this selective inhibition.

### Fluorescence imaging and binding interaction of B-QDs

**Fig. 5:**
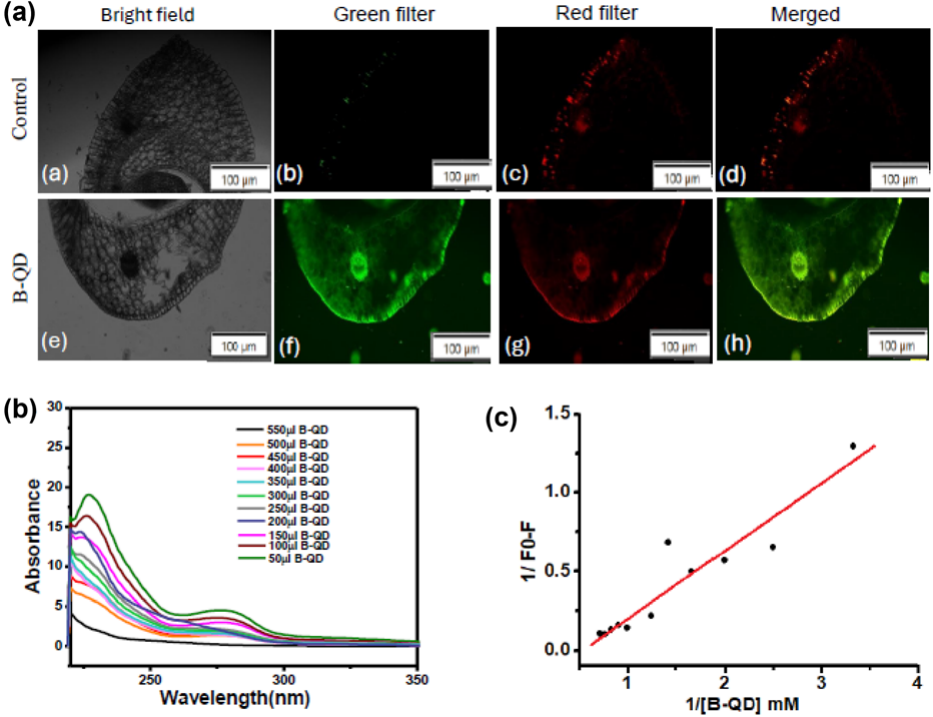
Light and fluorescence microscopy of a cross-section of the *V. mungo* root after treatment with B-QDs and water as the control, using both green and red filters. The images were captured at 100× magnification (a). UV-vis absorption spectra of pectic acid (500 µM) titrated with increasing concentrations (50–500 µM) of B-QDs (b). Benesi–Hildebrand plot (1/(*F*_0_ − *F*) *vs.* 1/[B-QD]) derived from the titration data, showing excellent linearity (c).

The distinct localization of B-QDs in the root tissues was shown by fluorescence imaging (Fig. 5a). The effective uptake and dispersion of the nanodots into root cells was confirmed by the bright-green and red fluorescence signals in the root sections of the treated group, which were significantly absent in the untreated/control group. The merged image showed consistent B-QD accumulation along the cortical and epidermal cell layers, indicating effective internalization and transport in root tissues. These results demonstrate that B-QDs could enter plant roots, mostly localize in the primary cell wall of plant cells, and disperse in a systematic manner, which will promote growth.

Pectic acid (a major constituent of primary cell walls) was chosen for the interaction study with B-QDs. The UV-vis absorption spectra of pectic acid (Fig. 5b) revealed a concentration-dependent drop in absorbance with increasing concentration of B-QDs. This hypochromic response indicates that the hydroxyl/carboxyl moieties of pectic acid and the surface functional groups of B-QDs may bind strongly and create a complex, which is probably caused by electrostatic attraction and hydrogen bonds. The lack of notable spectral shifts suggests that the binding quenches the nanodots' optical response through nonradiative pathways rather than significantly changing their core structure. Excellent linearity was shown by the Benesi–Hildebrand plot (1/(*F*_0_ − *F*) *vs.* 1/[B-QDs]), indicating the creation of a stable ground-state complex (Fig. 5c). The potential importance of pectic acid in regulating the stability, surface reactivity, and functional performance of B-QDs is highlighted by this strong binding behaviour. This is especially pertinent to the use of B-QDs in biological and agricultural systems. Therefore, B-QDs contribute to cell wall formation and strengthen cell adhesion and wall integrity. Several other nanoparticles have also been reported as promising candidates for efficient wastewater and soil remediation through pollutant detection, adsorption, and catalytic degradation owing to their unique physicochemical properties and high surface-to-volume ratios.^34,35^

**Fig. 6:**
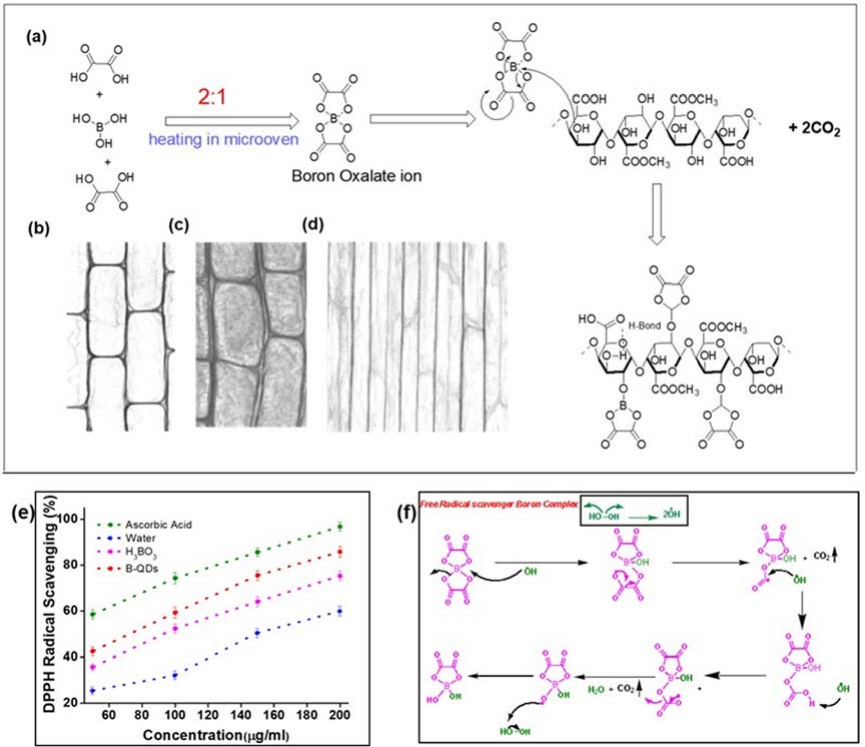
Schematic of boron-oxalate formation during B-QD synthesis and possible interaction with pectic acid (a). Parenchyma cells with thin primary walls and distinct intercellular spaces (b), collenchyma cells with unevenly thickened corners providing flexibility and mechanical support (c), and sclerenchyma fibres with thick, lignified secondary walls contributing to structural rigidity (d). Free radical scavenging activity-treated seeds following the DPPH assay; data are represented in comparison to the control ascorbic acid (e) and the mechanism of free radical scavenging activity (f).

It is obvious that B-QDs bind with pectic acid, and the boron atom is highly electron-deficient (a Lewis acid). The boron centre shows affinity for two adjacent hydroxyl groups (–OH) on two different sugar chains of pectic acid. This coordination forms a tight, cyclic ester-like complex, effectively cross-linking the polymer chains (Fig. 6a). By binding to these hydroxyl groups, the boron atom polarizes and weakens the C–O bonds. This Lewis acid catalysis makes the sugar carbons more susceptible to nucleophilic attack. Thus, B-QDs act as a transient, catalytic template. They bind pectic acid chains and organize them spatially to yield a mixture of valuable cyclic carbohydrate derivatives. Three distinct cell types, particularly parenchyma primary wall (Fig. 6b), collenchyma (Fig. 6c) and secondary wall sclerenchyma (fibres) (Fig. 6d), have pectic acid as the major constituent, and B-QDs are involved in making a solid and rigid structure.

Seedling extracts exhibited a clear dose-dependent increase in free radical scavenging activity (Fig. 6e); ascorbic acid, used as the control, showed the maximum inhibition at 200 µg mL^−1^. Water-treated controls displayed the lowest activity (59.8% ± 2.1% at 200 µg mL^−1^), while boric acid treatment improved radical scavenging (75.3% ± 2.2%). Notably, B-QD-treated seedlings achieved 85.8% ± 2.3% inhibition. These results demonstrate that the DPPH assay directly indicates the free radical scavenging capacity of extracts. Higher inhibition reflects stronger electron or hydrogen donation to neutralize the stable DPPH radical (Fig. 6f), reducing it from purple to yellow. The enhanced radical scavenging activity exhibited by B-QDs suggests that either quantum dot-mediated boron delivery may improve the antioxidant potential or through stimulation of endogenous antioxidant metabolites and/or direct surface-mediated reactive oxygen species quenching. This improved antioxidant capacity may consequently contribute to enhanced seed germination and plant growth.

## Conclusions

This study demonstrates that boron-doped carbon quantum dots provide dual benefits to plants by enhancing growth while simultaneously offering protection against pathogenic bacteria. Compared to conventional boron sources, B-QDs exhibit superior uptake, improved germination rates, and significant stimulation of root development. Their strong interaction with pectic acid and ability to penetrate root tissues highlight their mechanistic role in promoting cell wall dynamics and nutrient assimilation. Antimicrobial evaluations confirm the selective suppression of harmful phytopathogens alongside antibiofilm activity, underscoring their promise as environmentally friendly crop protectants. Importantly, B-QDs spare beneficial soil microbes, suggesting high potential for safe agricultural application. By integrating nutrient delivery with disease resistance, B-QDs represent an innovative quantum nanofertilizer platform that advances sustainable farming practices and reduces reliance on excessive agrochemicals.

## Experimental

### Synthesis of QDs

We synthesized boron-doped carbon quantum dots (B-QDs) using a microwave-assisted approach with boric acid and oxalic acid as precursors. A solution of 10 mM boric acid and 20 mM oxalic acid in 20 mL of deionized water was stirred continuously and then irradiated in a borosilicate container at 700 W for a total of 8 minutes in 2 minute cycles.^12^ During this process, the color of the solution typically changed from colourless to slightly light brown, indicating the formation of quantum dots. After microwave treatment, the solution was centrifuged at 15 000 rpm for 20 minutes, and the supernatant contained the B-QDs. These B-QDs were further characterized using UV-vis spectroscopy, XPS, and TEM analyses.

### UV-vis spectroscopy and band gap estimation *via* Tauc plots

The synthesised B-QDs were examined optically using a Lab India UV3200 UV-visible spectrophotometer.^13^ After being suitably diluted in deionised water, absorbance spectra in the 200–800 nm wavelength range with a pathlength of 1 cm were recorded using deionised water as a reference. To find the optical band gap, a Tauc plot was made using the absorbance values (*αhν*)^*n*^ = *A*(*hν* − *E*_g_), where *A* is a constant, *hν* is the photon energy, *E*_g_ is the optical band gap, *α* is the absorption coefficient, and *n* depends on the kind of electronic transition. For straight permitted transitions, *n* = 2 was selected. The absorption coefficient, *α*, from the absorbance (*A*) was calculated as follows: *α* = (2.303 × *A*)/*d*, where *d* is the cuvette's path length (1 cm). To determine the band gap energy, *E*_g_, the quantity (*αhν*)^2^ was plotted against *hν*, and the linear part of the curve was extrapolated to the *x*-axis.

### Transmission electron microscopy (TEM)

Using TEM on a JEOL JEM-2100 electron microscope running at an accelerating voltage of 200 kV, the shape and particle size of B-QDs were investigated.^13^ A 2 µL drop of the aqueous B-QD solution was applied on a copper grid coated with carbon to prepare the sample, and it was then allowed to air-dry at room temperature.

### XPS analysis

A PHI 5000 VERSA PROBE III instrument manufactured by ULVAC PHI (Physical Electronics), USA, was used to measure the XPS spectra.^14^ To lessen the effects of charging, a charge neutraliser was utilised during the measurement. The adventitious C 1s peak at 284.8 eV was used as the reference to calibrate the binding energy scale. Gaussian–Lorentzian peak morphologies with Shirley background correction were used for peak deconvolution.

### Fluorescence excitation and emission spectroscopy

The fluorescence spectra of B-QDs at room temperature were recorded using a spectrofluorometer (PerkinElmer LS55 or comparable). After being rapidly sonicated and dispersed in deionised water at a concentration of 0.1 mg mL^−1^, B-QDs were transferred to a 1 cm-long quartz cuvette. Excitation spectra were produced by tracking emission at the maximum emission wavelength, and emission spectra were recorded at the identified excitation maximum.^14^

### Well diffusion assay

*Pseudomonas syringae* pv. tomato DC3000 was cultured on Luria–Bertani (LB) agar plates (tryptone = 10 g L^−1^, yeast extract = 5 g L^−1^, NaCl = 10 g L^−1^; 1.5% agar) at 28 °C. For assays, a single colony was inoculated into the LB broth and incubated overnight at 28 °C with shaking (200 rpm) until mid-logarithmic growth (OD_600_ ≈ 0.5). The culture was washed and resuspended in sterile 0.85% NaCl to ∼10^8^ CFU mL^−1^ (0.5 McFarland standard). LB agar plates were seeded with 100 µL of the bacterial suspension, spread uniformly, and allowed to dry before loading 50 µL of each test solution into sterile 6 mm wells.^15^ After prediffusion (30 min, room temperature), the plates were incubated at 28 °C for 24–48 h.

### Determination of the minimum inhibitory concentration (MIC)

The minimum inhibitory concentration (MIC) of B-QDs was determined following the Clinical and Laboratory Standards Institute (CLSI) guidelines.^15^ Briefly, a two-fold serial dilution of the compound was prepared in sterile 96-well microtiter plates using Mueller–Hinton broth (MHB). The rhizobium minimal medium (RMM) is used for *Rhizobium* bacteria and contained mannitol (10.0 mg mL^−1^); K_2_HPO_4_ (0.5 mg mL^−1^); KH_2_PO_4_ (0.2 mg mL^−1^); MgSO_4_·7H_2_O (0.2 mg mL^−1^); NaCl (0.1 mg mL^−1^); CaCl_2_ (0.05 mg mL^−1^); FeSO_4_·7H_2_O (0.001 mg mL^−1^); and trace amounts of manganese (Mn^2+^), zinc (Zn^2+^), and copper (Cu^2+^). The pH was adjusted to 6.8–7.0. The AB minimal medium was used for *Agrobacterium tumefaciens*. The medium consisted of K_2_HPO_4_ (3.0 mg mL^−1^); NaH_2_PO_4_ (1.0 mg mL^−1^); NH_4_Cl (1.0 mg mL^−1^); MgSO_4_·7H_2_O (0.2 mg mL^−1^); CaCl_2_·2H_2_O (0.01 mg mL^−1^); FeSO_4_·7H_2_O (0.005 mg mL^−1^) and trace amounts of MnCl_2_ (0.001 mg mL^−1^). The pH was adjusted to 7.0. The microbial inoculum was adjusted to a 0.5 McFarland standard (approximately 2 × 10^8^ CFU mL^−1^) and then further diluted to achieve a final concentration of ∼5 × 10^5^ CFU mL^−1^ in each well. Each well contained different concentrations of B-QDs and the medium, with a final volume of 200 µL per well. Positive control wells (inoculum without the compound), sterility control wells (media only), and standard antibiotic controls were inoculated. The plates were incubated at 37 °C for 16 hours under appropriate conditions. Following incubation, the wells were examined for visible microbial growth. The MIC was defined as the lowest concentration of the B-QDs that completely inhibited visible growth of the organism. All experiments were performed in triplicate, and results were interpreted according to the CLSI guidelines.

### Antibiofilm assay

The antibiofilm activity of the B-QDs against *Pseudomonas syringae* was evaluated using a 96-well microtiter plate crystal violet staining method.^13^*P. syringae* was cultured overnight and diluted to 1 : 100 in a Luria Broth (LB) medium after being adjusted to an optical density of 0.1 at 600 nm (about 1 × 10^8^ CFU mL^−1^). In a sterile flat-bottom 96-well plate, 100 µL of the B-QDs at different concentrations was combined with 100 µL of the bacterial suspension in each well. Both untreated bacteria (positive control) and the bacterial-free medium (negative control) were present in the control wells. For 48 hours, the plates were incubated at 37 °C without being shaken to promote the production of a biofilm. PBS was used to wash the wells three times in order to get rid of nonadherent cells after the planktonic cells were removed after incubation. The remaining biofilm was fixed with methanol for 15 minutes, air-dried, and stained with 0.1% (w/v) crystal violet for 15 minutes at room temperature. After washing with sterile distilled water to remove any remaining stain, 95% ethanol or 33% acetic acid was used to dissolve the bound dye. A microplate reader was used to detect the absorbance at 570 nm. The absorbance values of the treated and untreated control wells were compared in order to determine the percentage suppression of biofilm formation. All assays were performed in triplicate, and the data were analyzed statistically to determine the significance of biofilm inhibition.

### Seed germination assay

*Vigna mungo* seeds were surface-sterilised for 2 minutes with 0.1% HgCl_2_ and then completely rinsed with sterile distilled water. The seeds were then immersed for 12 hours in various concentrations of the investigated nutrients and their associated QDs, with deionised water serving as the sole control at a concentration of 1 µg mL^−1^. The treated seeds were placed on Petri dishes lined with wet filter paper and incubated for 3 days at room temperature (25 ± 2 °C) in a dark atmosphere.^16^ Three independent replicates (*n* = 50 seeds per group) were used to calculate the germination percentage.

### Fluorescence imaging of B-QD-treated roots

Clean roots treated with B-QDs (1 µg mL^−1^) were blotted dry using sterile filter paper and then cut with a sharp scalpel into uniform segments. Depending on the needs of the experiment, the portions were either processed or corrected right away.^17^ To prevent further contamination, every step was carried out in an aseptic environment. The distribution of B-QDs was visualised using fluorescence imaging using an Olympus epifluorescence microscope equipped with red and green filters. Images of three biological replicates were captured at a 10× magnification, and the fluorescence intensity was measured using ImageJ software.

### Root growth and morphometric analysis

*Vigna mungo* seedlings were cultivated and treated with deionised water (control) and a fixed concentration (1 µg mL^−1^) of B-QDs or H_3_BO_3_. Roots were analysed as the total root length and the number of roots. Calculations were made to determine the average fold changes in the root length and number compared to the control group for quantitative analysis.^18^

### UV-vis absorption spectroscopy and kinetics analysis

Absorption spectra were recorded on a UV-vis spectrophotometer (Lab India UV3200 UV-visible spectrophotometer) in the range of 200–800 nm using quartz cuvettes with a 1 cm path length. Pectic acid (fixed concentration of 500 µM) was titrated with increasing concentrations of B-QDs (50–500 µM), and spectra were collected after 10 min equilibration at room temperature. The absorbance values were baseline-corrected against the corresponding blanks. The interaction between B-QDs and pectic acid was quantified using the Benesi–Hildebrand equation.

### Free radical scavenging assay

Sterilized seeds were soaked for 12 h at room temperature with gentle agitation in the following treatment solutions: (i) sterile distilled water, (ii) boric acid (1 µg mL^−1^), or (iii) B-QDs (1 µg mL^−1^). 50–100 seeds were used per replicate. Treated seeds were subsequently transferred to moist sterile filter paper for 3 days. Seedlings were blotted dry and weighed (∼100 mg fresh weight per replicate). Tissues were ground in liquid nitrogen and extracted with methanol (80% v/v; 1 mL per 100 mg of the tissue). Homogenates were vortexed for 1 min, sonicated for 10 min, and incubated for 30 min at room temperature with gentle agitation. Extracts were centrifuged at 12 000×*g* for 10 min at 4 °C, and the resulting supernatants were collected. Extracts were diluted to final assay concentrations of 50, 100, 150, and 200 µg mL^−1^ (normalized with the protein concentration). l-ascorbic acid at an identical concentration served as the control. For each assay, 1.0 mL of the DPPH solution was combined with 1.0 mL of the extract (or 100 µL each in the 96-well plate format). The reaction mixtures were incubated at room temperature for 30 min in the dark, after which the absorbance was measured at 517 nm. Extract blanks (extract + methanol) were subtracted to correct for sample background. Radical scavenging activity was expressed as percentage inhibition:
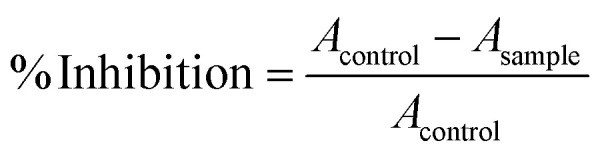


## Author contributions

SC, SKM, TKB performed the experiments, PB prepared some illustrations, SR, DP, GG and SMM supervised the work, and SMM and GG conceived the idea.

## Conflicts of interest

There are no conflicts to declare.

## Data Availability

Data are publicly available in the Science Data Bank under a-CC BY-NC 4.0 license at the following link: https://www.scidb.cn/en/anonymous/Vk5yTXoy and https://doi.org/10.57760/sciencedb.33615.
